# Quantitative outcomes of a type 2 single arm hybrid effectiveness implementation pilot study for hypertension-HIV integration in Botswana

**DOI:** 10.1186/s43058-024-00620-w

**Published:** 2024-07-22

**Authors:** Thato Moshomo, Tendani Gaolathe, Mareko Ramotsababa, Onkabetse Julia Molefe-Baikai, Edwin Mogaetsho, Evelyn Dintwa, Pooja Gala, Ponego Ponatshego, Laura M. Bogart, Nabila Youssouf, Khumo Seipone, Amelia E. Van Pelt, Kara Bennett, Shabbar Jaffar, Maliha Ilias, Veronica Tonwe, Kathleen Wirth Hurwitz, Kago Kebotsamang, Karen Steger-May, Lisa R. Hirschhorn, Mosepele Mosepele

**Affiliations:** 1 Department of Internal Medicine, Faculty of Medicine, University of Botswana, Gaborone, Botswana; 2 Botswana Harvard Health Partnership, Gaborone, Botswana; 3 Department of Medicine, NYU Langone Grossman School of Medicine, New York, NY USA; 4 RAND Corporation, Santa Monica, CA USA; 5 Charles R. Drew University of Medicine and Science, Los Angeles, CA USA; 6 Department of Clinical Research, Faculty of Infectious and Tropical Diseases, London School of Hygiene and Tropical Medicine, London, UK; 7 African Comprehensive HIV/AIDS Partnership (ACHAP), Gaborone, Botswana; 8 Department of Medical Social Sciences, Northwestern University, Chicago, IL USA; 9 Bennett Statistical Consulting, Inc, Ballston Lake, NY USA; 10 Institute for Global Health, University College London, London, UK; 11 Department of Health and Human Services, Center for Translation Research and Implementation Science, National Heart, Lung and Blood Institute, National Institutes of Health, Bethesda, MD USA; 12 NoviSci, Inc., a Target RWE Company, Durham, NC USA; 13 Department of Statistics, Faculty of Social Sciences, University of Botswana, , Gaborone, Botswana; 14 The Center for Biostatistics and Data Science at Washington University School of Medicine’s Institute for Informatics, Data Science and Biostatistics, St. Louis, USA

**Keywords:** Hypertension, HIV, Integration, Implementation, Pilot study, Botswana, Electronic health records, Noncommunicable diseases, Hybrid effectiveness-implementation

## Abstract

**Background:**

Successful HIV treatment programs have turned HIV into a chronic condition, but noncommunicable diseases such as hypertension jeopardize this progress. Hypertension control rates among people with HIV (PWH) are low owing to gaps in patient awareness, diagnosis, effective treatment, and management of both conditions at separate clinic visits. Integrated management, such as in our study, InterCARE, can enhance HIV-hypertension integration and blood pressure (BP) control.

**Methods:**

Our pilot study was conducted in two Botswana HIV clinics between October 2021 and November 2022. Based on our formative work, we adopted three main strategies; Health worker training on HTN/cardiovascular disease (CVD) management, adaptation of HIV Electronic Health Record (EHR) for HTN/CVD care, and use of treatment partners to support PWH with hypertension for implementation. We employed the Reach, Effectiveness, Adoption, Implementation, Maintenance (RE-AIM) framework to assess implementation effectiveness and outcomes for BP control at baseline, 6 and 12 months. HIV viral load (VL) suppression was also measured to assess impact of integration on HIV care.

**Results:**

We enrolled 290 participants; 35 (12.1%) were lost to follow-up, leaving 255 (87.9%) at 12-months. Median age was 54 years (IQR 46–62), and 77.2% were females. Our interventions significantly improved BP control to < 140/90 mmHg (or < 130/80 mmHg if diagnosis of diabetes or chronic kidney disease), from 137/290 participants, 47.2% at baseline to 206/290 participants, 71.0%, at 12 months (*p* < 0.001). Among targeted providers, 94.7% received training, with an associated significant increase in counseling on exercise, diet, and medication (all *p* < 0.001) but EHR use for BP medication prescribing and cardiovascular risk factor evaluation showed no adoption. In the intention-to-treat analysis, HIV VL suppression at 12 months decreased (85.5% vs 93.8%, *p* = 0.002) due to loss to follow-up but the per protocol analysis showed no difference in VL suppression between baseline and 12 months (97.3% vs 93.3%, *p* = 0.060).

**Conclusion:**

The InterCARE pilot study demonstrated that low-cost practical support measures involving the integration of HIV and hypertension/CVD management could lead to improvements in BP control. These results support the need for a large implementation and effectiveness trial.

**Trial registration:**

ClinicalTrials.gov NCT05414526. Registered 18th May 2022.

**Supplementary Information:**

The online version contains supplementary material available at 10.1186/s43058-024-00620-w.

Contributions to the literature

- Integrating hypertension care into existing HIV services in Botswana led to significant improvement in BP control, from 47% (baseline) to 71% (12 months) participants with controlled BP.
- Community-based treatment partners were willing to support adults with comorbid HIV-hypertension. However, further research is needed to better understand how to optimize the role of treatment partners under a differentiated service delivery strategy.
- Low uptake of EHR documentation in HIV-hypertension care contributes to a lack of data on key non-communicable disease indicators among PWH.
- There is no early evidence of adverse effects of HIV-hypertension integration on HIV viral load outcomes.

## Background

Successful HIV treatment programs have led to longer lives for people with HIV (PWH) in low- and middle-income countries (LMIC), but non-communicable diseases (NCDs) now pose a major threat, potentially reversing progress in reducing HIV-related mortality [1–5]. Globally, approximately 27% of PWH on antiretroviral treatment (ART) are living with hypertension (HTN) [5]. While the majority of PWH and HTN are undiagnosed [6], only 25% of those diagnosed are receiving HTN treatment [5], leading to suboptimal control and its associated cardiovascular disease (CVD) risks such as stroke, coronary artery disease, and kidney disease [7–9]. CVD among PWH has tripled over the last two decades and is now responsible for 2.6 million disability adjusted life years (DALYs) annually [4].

In Botswana, a setting with a young population (median age 24 years [10], high HIV prevalence (20.8% among those 15–64 years of age) [11], and high HTN prevalence (26.8% among those 15–64 years of age) [12], the hypertension care cascade (aware of diagnosis, on treatment, controlled) falls significantly short of national [13] and global targets [14]. Botswana’s national target is 80% BP control by 2025 [13] among adults, and the global target is at least 75% BP control by 2050 [14]. A recently published local study of 766 participants with HTN, including 213 PWH, reported that only 40.0% participants were previously aware of their HTN diagnosis, and of those aware, only 30.4% of PWH were receiving HTN treatment, with a control rate of 41.7% [12].

This hypertension cascade of care contrasts with Botswana’s success in the HIV cascade of care, in which higher proportions of PWH are aware of their status, receiving ongoing ART, and have achieved viral suppression, which corresponds to the UNAIDS 95–95-95 targets [15]. This success can be attributed to factors such as specialized HIV clinics [16, 17], well-trained staff [18], task shifting [19], the existence of a locally designed national electronic health records system (EHR) [20], free access to ART [17, 21], and use of treatment partners [22]. Thus, successful care is possible, but how to leverage these effective strategies to improve the HTN cascade of care in the same setting is not understood.

To address this gap, we developed a strategy called ‘INTEgrating hypeRtension and cardiovascular diseases CARE into existing HIV services package in Botswana’ (InterCARE), that utilizes adapted Electronic Health Record (EHR), provider training, and treatment partners for adults with a dual diagnosis of HIV and HTN. Our primary aim was to develop a set of implementation strategies for integrated HIV-CVD care within an existing HIV care platform and then to conduct a pilot study to explore outcomes using the Reach, Effectiveness, Adoption, Implementation, Maintenance (RE-AIM) framework [23], to inform adaptation for broader testing and scale up. The study hypothesis was that integrating HTN care within an existing HIV care platform is feasible, acceptable, and associated with early improvement in the hypertension cascade of care. We also assessed the impact of integration on retention in HIV care and the effects on HIV viral load suppression.

## Methods

### Interventions and strategies

Initially, a mixed-method formative research approach was employed, using the Consolidated Framework for Implementation Research (CFIR) [24, 25] to assess contextual factors at the national, facility, and community/patient level influencing the presence of gaps in hypertension cascade of care at HIV clinics (reported separately in detail) [26]. Prior to our pilot study, formative work was conducted to measure gaps in care, facility readiness, and factors affecting the implementation of HIV-HTN/CVD integration. This was done with a goal of leveraging on the key strategies that facilitated Botswana’s highly successful national ART program despite the resource limitations. A detailed description of this formative work is reported separately [26]. Briefly, the formative work involved a mixed method approach: surveys by all relevant stakeholders (health care workers, PWH, treatment partners, community members) and key informant interviews on a sample of the surveyed stakeholders, at baseline and end of the study. The significant barriers identified included limitations in available resources, structural infrastructure (e.g., levels of staffing), and access to knowledge and information (REF). To address the identified structural barriers, such as staffing levels, workload and access to knowledge among healthcare providers, a comprehensive provider training on HTN/CVD, which included one-on-one coaching and practice facilitation, was used, see Table 1.

**Table 1:** Summary of the implementation strategies designed to support HIV/HTN care integration and their related outcome measures

Strategy	Components	Outcome measure
Healthcare Provider HTN/CVD curriculum & Training	• Adaptation of an old MoH HTN management protocol, Botswana primary care guidelines [27] plus the Centers for Disease Control and Prevention (CDC, USA) Hypertension Management Curriculum [28] • Workshops materials developed using principles of interprofessional education • Online course of ten modules with pre- and post-test assessments • Post-workshop training was followed with on-site clinic mentoring through coaching and practice facilitation	• absolute difference in systolic and diastolic BP between baseline and 12-months visit • the proportion of participants who scored > 70% on the HDFQ • proportion of those who received CVD risk factor counselling on anti-HTN medication, healthy diet and appropriate physical activity levels • proportion prescribed guideline concordant BP medication (as per Botswana 2017 Primary Care Guideline and/or major publication on management of BP among Black Africans
EHR adaptation	• Adaption of a locally developed MoH EHR system referred to as “PIMS”, to include the following ◦ Addition of reminders to record BP and weight; screen and counsel patients about physical activity, salt intake, and weight management; obtain a lipid profile if on protease inhibitor; obtain a lipid profile if > 50 years old and none is documented from the preceding 5 years ◦ EHR programming so that CVD risk prediction can occur automatically in the EHR when all the required input variables from the preceding 24 months are available	• proportion of clinic encounters in EHR (at the 12 months visit) where anti-HTN medications are prescribed if indicated, extracted from the HIV clinic EHR (E-prescribing) • proportion of PWH and HTN with 10-year CVD risk documented in EHR (E-CVDRF eval) • proportion with BP documented in EHR
Treatment Partners	• Participants were paired with self-selected community-based treatment partners (from patient’s social network) ◦ Treatment partners practiced role-plays with trainers/counselors informed by motivational interviewing strategies • Treatment partners trained on HTN/CVD care • Role of treatment partner: ◦ communicate at least weekly with participant to provide counselling ◦ support on medication adherence and attending clinic appointments, HTN knowledge, and healthy diet and exercise	• proportion of participants with a treatment partner • proportion of treatment partners trained per protocol • proportion providing support with the same frequency as expected

The main primary effectiveness quantitative outcome of change from baseline to 12 months in the proportion of PWH with a diagnosis of HTN and on medication with controlled blood pressure is a due to a composite strategies from HTN/CVD training and treatment partner support

*MoH* Ministry of Health, *HTN* Hypertension, *HER* Electronic health record, *CVD* Cardiovascular disease, *PIMS* Patient integrated management system

Based on the results of this work [26], we also adopted the two other strategies of; adaptation of HIV EHR for HTN/CVD care, and use of treatment partners to support PWH with HTN for implementation (See Table 1 and supplementary table 1).

First, adaptation of the EHR was done in collaboration with the Ministry of Health (MoH). EHR use was already ongoing at the clinic sites which were used to pilot InterCARE. This adaptation included the addition of reminders to record BP and weight; screen and counsel patients about lifestyle modifications; obtain a lipid profile if on protease inhibitor or if > 50 years old and none is documented from the preceding 5 years. Additionally, EHR was programmed so that CVD risk prediction can occur (at 0, 6 and 12 months) automatically in the EHR when all the required input variables from the preceding 24 months are available. Our EHR adaptations (specific to hypertension) were made on September 27th 2021, and the adapted EHR was used for the first participant (with HIV and hypertension) on October 4th 2021.

Second, health provider training was conducted by the study team at the 2 selected clinics using materials jointly developed with the MOH. This started on September 27th 2021. When clinic staff turnover exceeded 50%, healthcare provider re-training was conducted. The study doctor and nurses were also available to coach healthcare providers on HTN management throughout the 12 months of the pilot period. Providers received training on HTN/CVD integrated care, including counselling on lifestyle modification.

Third, patient-selected treatment partners were contacted within 4 weeks of index participant enrollment if the enrolment BP was uncontrolled, or up to 12 weeks if the BP was controlled. Treatment partners were educated about high blood pressure in person, through video and written materials and capacitated with modified motivational interviewing skills to support patients. The first treatment partner was enrolled on November 8th 2021.

We followed the Standards Reporting for Implementation Studies Checklist (StaRI) [29] in writing the manuscript.

### Pilot study

We pilot tested this set of three multi-component implementation strategies using a single arm pre-post type 2 hybrid effectiveness implementation pilot study at one small rural clinic (< 500 PWH) in southwest Botswana and one large urban/peri-urban HIV clinic (> 2,000 PWH) in northeast of Botswana. The two clinics were selected because they were geographically distant (to minimize contamination) and their size as well as staff composition was representative of the majority of HIV clinics in Botswana. Additionally, they were representative of both rural and periurban settings, as well as a small and large HIV clinic in Botswana.

### Eligibility criteria

Inclusion criteria for patients were age 20–75 years old; being on ART and in care at the site; and with a confirmed diagnosis of HTN or elevated SBP (≥ 140 mmHg) and/or elevated DBP (≥ 90 mmHg). Cutoffs of ≥ 130 or ≥ 80 mmHg were used if patients had a previous diagnosis of Diabetes Mellitus or chronic kidney disease at the time of screening.

The exclusion criterion for patients included any medical diagnosis (e.g., severe memory impairment) in their medical records that would make it difficult for them to comprehend planned education and counseling.

The inclusion criteria for treatment partners were age 18 years and above and were selected by the index participant. Participants could choose another adult with or without HIV as their treatment partner. Exclusion criteria for the treatment partners included any cognitive diagnosis that would make it difficult to serve as an effective treatment partner.

The inclusion criteria for the providers were any nurse or medical doctor who provided direct HIV care during the designated HIV clinic days at the two study sites.

### Recruitment

Participant recruitment and 12-month follow-up took place between October 2021 and November 2022. A convenience sampling technique was used for participant selection. A trained study nurse or research assistant prescreened (on pre-HIV clinic visit day) patient charts and placed study fliers in eligible patients’ charts. The selected patients were then told about the pilot study during their HIV clinic visit. Those who were prescreened negative were also approached on the day of the clinic to assess whether their clinic day BP readings or review of any other medical records in their possession made them eligible for participation (allowing for new HTN diagnosis and merging of separate [non-HIV clinic records] clinical details about patients’ BP status). After enrollment, patients were told to nominate a treatment partner. The study team then contacted the treatment partner via telephone and arranged meetings to screen and enroll the treatment partner.

### Study Population and procedures

All PLWH and hypertension eligible and enrolled in the pilot phase of our study underwent a baseline study visit with trained research assistants that included collection of recorded anthropometric data and self-reported socio-demographic, economic, and clinical data. Participant health records were accessed by research assistants to collect co-morbidity, prescription, and laboratory data for enrolled participants.

In terms of blood pressure assessment, qualified healthcare workers at the different participating clinics received training from the study team on correct measurement of blood pressure, among other CVD risk factors, using clinic provided equipment (varied across sites- from automated to manual BP machines and different models). The healthcare workers used three arm blood pressure readings using either an automated or manual blood pressure cuff as recommended [30]. An average of the three blood pressure readings were used were obtained and subsequently used by our team in the analysis.

### Data collection

Sources of data for both effectiveness and implementation outcomes are shown in Table 2. Participant related data was collected from available electronic and paper records at baseline, and at 6 months, and 12 months within a 3-month window at the second and third timepoint. All data was stored in REDCap© [31, 32].

**Table 2:** Secondary effectiveness and implementation quantitative outcomes

Secondary outcomes		Sources
Effectiveness	• absolute difference in systolic and diastolic BP between baseline and 12-months visit • the proportion of participants who scored > 70% on the HDFQ	• Modified EHR, DCF
Reach	• proportion of eligible PWH who agreed to be enrolled into InterCARE among those who met inclusion criteria	• Modified EHR, DCF
Adoption	• proportion of PWH and HTN with 10-year CVD risk documented in EHR (E-CVDRF eval) • proportion with BP documented in EHR • proportion of those who received CVD risk factor counselling on anti-HTN medication, healthy diet and appropriate physical activity levels	• Modified EHR, DCF
Fidelity	• proportion prescribed guideline concordant BP medication (as per Botswana 2017 Primary Care Guideline [27, 33] and/or major publication on management of BP among black Africans [34] • proportion of treatment partners trained per protocol • proportion providing support with the same frequency as expected	• DCF, electronic trial logs
Exploratory secondary outcomes		
Fidelity/ Adoption	• proportion of participants with a treatment partner	• Modified EHR, DCF

*HER* Electronic Health Record, *BP* Blood Pressure, *HDFQ* Heart Disease Fact Questionnaire: *CVD* Cardiovascular Disease, *E-CVDRF eval* Electronic-Cardiovascular Disease Risk Factor Evaluation, *PWH* People with HIV, *DCF* Data Collection Form

### Outcomes

Our main primary effectiveness quantitative outcome was the change from baseline to 12 months in the proportion of PWH with a diagnosis of HTN and on medication with controlled blood pressure defined as BP < 140/90 mmHg, or < 130/80 mmHg for those with comorbid type 2 diabetes mellitus or Chronic Kidney Disease (CKD) [27, 35]. The RE-AIM framework was used as noted above and, our primary implementation quantitative outcome was adoption, defined as the proportion of clinic encounters in EHR (at the 12 months visit) where anti-HTN medications are prescribed if indicated, extracted from the HIV clinic EHR (E-prescribing). Our combined secondary effectiveness and implementation quantitative outcomes are shown in Table 2.

We monitored unintended effects/harms of InterCARE on the HIV program by documenting change in study cohort HIV viral load at baseline versus at 12 months. All efforts were made to find HIV viral loads for all those who discontinued participation in InterCARE but remained alive and their records could be accessed electronically through the Patient Intergrated Management System (PIMS).

### Statistical analysis

Demographic and clinical characteristics were summarized by performing a standard descriptive analysis (percentages, median and IQR) for skewed data or means (standard deviation) for normally distributed data. Chi-square tests for independence were used to assess the association between categorical variables, whereas the Wilcoxon rank sum test/Kruskal–Wallis test was used to compare the medians of the continuous variables, as appropriate. A summary of the hypertension care cascade defined as, the proportion aware of their hypertension diagnosis; then the proportion of those with hypertension on medications; and the proportion of those taking medications who are controlled, was compared from baseline versus at 12 months. In the quantitative primary and secondary outcome analysis, an intention to treat approach was used. In the sensitivity analysis, those who discontinued due to adverse events unrelated to the pilot study, plus those who discontinued because they had to transfer HIV care from the pilot sites, were excluded in a modified intent to treat analysis. Finally, an analysis including only those who attended the final study visit (per protocol analysis) was conducted to fully assess the outcomes of the implementation strategies. All analysis were performed using R-statistical software, v4.3.0© [2023].

## Results

**Fig. 1:**
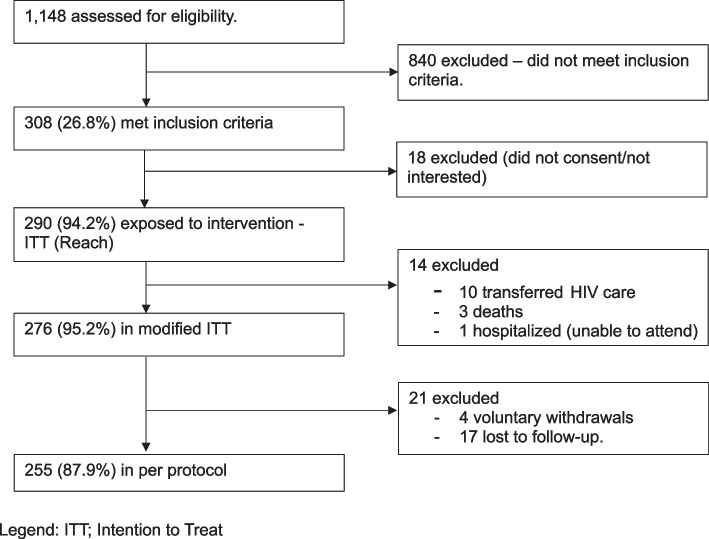
InterCARE study flow diagram, Legend: ITT; Intention to Treat

After screening 1,148 PWH, 308 (26.8%) met inclusion criteria, from whom 290 (94.2%) were enrolled (50 at the small and 240 at the large HIV clinics). Subsequently, 14 participants were excluded due to transfer of HIV care to other clinics, *n* = 10, death, *n* = 3, and inability to attend follow-up due to hospitalization, *n* = 1, both unrelated to the study. A further 21 participants either withdrew from the study (*n* = 4) or were lost to follow-up (*n* = 17); thus, 255 (87.9%) participants completed the 12-month follow-up (Fig. 1 and Table 3).

**Table 3:** Socio-demographic and clinical characteristics of InterCARE participants at baseline and 12-months

	Total Baseline (*n* = 290)	Total 12 months (*n* = 255)
**Age**		
Median (IQR)	54 (46—62)	56 (48—63)
**Sex**		
Females	224 (77.2%)	200 (78.4%)
**Education Level**		
Less than primary	62 (21.4%)	N/A
Primary	119 (41.0%)	N/A
Junior Secondary	83 (28.6%)	N/A
Senior Secondary	12 (4.1%)	N/A
AS/A-Level^a^	1 (0.3%)	N/A
Tertiary	13 (4.5%)	N/A
**Employment Status**		
Employed	104 (35.9%)	76 (29.8%)
Unemployed	186 (64.1%)	179 (70.2%)
**Employment Type**		
Formal	36 (34.6%)	28 (36.8%)
Informal	68 (65.4%)	48 (63.2%)
**Monthly Income, in Pula**		
Missing	9 (3.10%)	0
No Income	66 (22.8%)	86 (33.7%)
< 1000	139 (47.9%)	112 (43.9%)
1000–5000	67 (23.1%)	51 (20.0%)
5001–10,000	4 (1.4%)	3 (1.2%)
> 10,000	5 (1.7%)	3 (1.2%)
**Any Tobacco Use**	18 (6.2%)	13 (5.1%)
Smoking (Current)	25 (8.6%)	5 (2.0%)
**Number of Co-morbidities (besides HIV & HTN)**		
0	260 (89.7%)	222 (87.1%)
1	24 (8.3%)	29 (11.4%)
2	6 (2.1%)	4 (1.6%)
**Hypertension medication**		
Current	242 (83.4%)	231 (90.6%)
**Body mass index (BMI)**		
Underweight	20 (6.9%)	15 (5.9%)
Normal Weight	101 (34.8%)	86 (33.7%)
Overweight	95 (32.8%)	78 (30.6%)
Obese	74 (25.5%)	76 (29.8%)

^a^AS/A level; British General Certificate of Education A level – equivalent to American High School Diploma

### Baseline characteristics and 12 months follow-up

At baseline, the median age of those enrolled was 54 years (IQR 46–62), most participants were female (*N* = 224, 77.2%), approximately two thirds had; primary education or lower (62.4%), were unemployed (*N* = 186, 64.1%), and had a high BMI (*N* = 169, 58.3%) (Table 3). Most were on BP medications (*N* = 242, 83.4%),

The smaller rural clinic had more unemployed participants at baseline (80.0%) compared to the larger urban clinic (60.8%), (*p* = 0.016), and after 12 months, a significant income disparity emerged, with most individuals in the smaller clinic having no income, while the larger clinic had higher income (*p* < 0.001) (Supplementary Table 2). Additionally, the smaller clinic had more participants without comorbidities other than HIV and HTN (98%) compared to the larger clinic (84.4%), (*p* = 0.029) (Supplementary Table 2).

The HTN care cascade (Table 4); on treatment, controlled BP, on anti-HTN and controlled BP showed no change in those on treatment but an improvement in those with controlled BP, and those with controlled BP on HTN medications. Furthermore, the HTN care cascade was better in the smaller clinic (94.0%, 88.0%, 84.0%) than the larger clinic (76.7%, 67.5%, 61.3%), respectively, (all *p* < 0.001) (Supplementary Table 3).

**Table 4:** Hypertension care cascade

	**Baseline** **(*n* = 290)**	**12 months (*n* = 255)**^**a**^	**95% CI**^c^	***p*-values**
Aware of diagnosis	268 (92.4%)	n/a		
On Treatment	242 (83.4%)	231 (79.7%)	-10.4, 2.86	0.284
Controlled BP	137 (47.2%)	206 (71.0%)	**15.7, 31.9**	** < 0.001**
Taking HTN Medications and Controlled BP^b^	133 (55.0%)	189 (81.8%)	**11.0, 27.6**	** < 0.001**

^a^The 12 month follow-up proportions are based on *n* = 290 using the Intent to Treat analysis

^b^The proportions are based on the total number of participants on HTN medications

^c^95% CI of the Difference at 12 months vs baseline

### Quantitative primary and secondary outcomes (Intention-to-treat analysis)

As shown in Table 5, good effectiveness was seen with improved blood pressure control from 137/290 participants (47.2%) to 206/290 (71.0%), associated with a 10.3 mmHg and 8.5 mmHg drop in systolic and diastolic blood pressures respectively (Table 5), (all *p* < 0.001). In the sensitivity analyses (secondary analysis to assess the sensitivity of the results to variations in loss to follow up), blood pressure control was 74.6% in the modified intention-to treat and increased to 81.0% in the per-protocol analysis relative to 47.2% control at baseline.

**Table 5:** InterCARE primary and secondary RE-AIM outcomes

Primary Outcomes, n (%)				
	Baseline (*n* = 290)	**12 months**^**c**^	95% CI^b^	*p*-value
**Effectiveness**				
% controlled BP^a^	137(47.2)	206(71.0)	15.7, 31.9	< 0.001
**Implementation**				
% e-prescribing	41(14)	0(0)	-18.5, -9.8	< 0.001
**Secondary Outcomes, n (%)**				
**Reach**				
% eligible PWH enrolled into InterCARE	308 (26.8)	n/a	n/a	n/a
**Effectiveness**				
Mean SBP (mmHg)	134.6	124.3	-12.9, -7.6	< 0.001
Mean DBP (mmHg)	87.9	79.4	-10.3, -6.6	< 0.001
% pass HDFQ	111 (38.0)	233 (80.0)	34.5, 49.6	< 0.001
**Adoption**				
% e-CVDRF eval	0(0)	0(0)	n/a	n/a
% with BP documented in EHR	253 (87.0)	0 (0)	-91.4, -83.1	< 0.001
Medication counselling	0(0)	176 (61.0)	54.7, 66.7	< 0.001
Diet counselling	0(0)	237 (82.0)	76.9, 86.5	< 0.001
Exercise counselling	0(0)	225 (78.0)	72.4, 82.7	< 0.001
**Fidelity**				
% Correct BP medications^d^	235 (81.0)	224 (77.0)	-10.7, 3.2	0.307
%Treatment partners trained	290 (100)	n/a	n/a	n/a
% Treatment partners providing support	290 (100)	255 (88.0)	-16.2, -8.0	< 0.001
%Targeted providers who received training	18 (94.7)	n/a	n/a	n/a

*HER* Electronic Health Record, *BP* Blood Pressure, *HDFQ* Heart Disease Fact Questionnaire, *CVD* Cardiovascular Disease, *E-CVDRF eval* Electronic-Cardiovascular Disease Risk Factor Evaluation, *PWH* People with HIV, *DCF* Data Collection Form

^a^BP documented in paper chart

^b^95% CI of the Difference at 12 months vs baseline

^c^The 12 month follow-up proportions are based on *n* = 290 since we adopted ITT analysis

^d^Correct BP medications: As per evidence based guidelines [28] or recommendations [34]

There was a significant difference in DBP between the small and large clinic both at baseline and 12 months, *p* = 0.004. All the participants in the small clinic had a pass in the HDFQ compared to 76.3% in the large clinic, *p* < 0.001.

Eighteen of 19 (94.7%) healthcare providers completed training to offer integrated HIV/HTN care across the two study sites (one targeted provider was unavailable for training) however, there were no clinic encounters during which anti-HTN medications prescribed were documented in EHR (E-prescribing) (primary implementation outcome). Counseling increased significantly for exercise, diet, and medication (all *p* < 0.001) (Table 5), particularly in the small clinic at 88.0%, 100% and 98.0%, compared to the large clinic, 55.0%, 77.9% and 73.3%, respectively (all *p* < 0.001) (Supplementary Table 4). Lastly, the smaller clinic had a greater fidelity of correct BP medications than the larger clinic, 90% vs 74.6% (*p* = 0.029) (Supplementary Table 4). Although there seems to be fewer treatment partners providing support at 12 months follow up, 88%, it must be noted that this is based on ITT, thus in the per protocol analysis, there are 100% treatment partners providing support at 12 months follow up. Additional quantitative RE-AIM outcomes are presented in Table 5, and in supplementary tables 4.

### Unintended effects/harm of InterCARE on the HIV program

The rate of viral load suppression at 12 months, compared to baseline, was significantly lower in the intention to treat analysis (85.5% vs. 93.8%, 95% CI of the difference -13.5—-3.0, *p* = 0.002). Aside from the 4 participants who discontinued the study due to death/hospitalization, 31 of the 35 (12.1%) participants’ medical records were reviewed with only 4 found to have HIV viral load from a different clinic after leaving the study, all of which were undetectable. For the remaining 27 participants who discontinued, none had documented viral load data for at least 8 months as of June 2023, indicating they were no longer receiving recommended HIV monitoring. Therefore, in the per protocol analysis, the rate of viral suppression was similar, at 12-months, 248/255 (97.3%) versus baseline, 238/255 (93.3%), 95% CI of the difference: -0.3–7.3, *p* = 0.060.

## Discussion

Our single arm pilot study in a high HIV-HTN prevalence setting, in Botswana demonstrates the effectiveness of integrating HTN into HIV care in small and large HIV clinics for control of HTN. While successful, with a 24% absolute increase in BP control among PWH, from baseline to 12 months, it highlighted the need to enhance strategy adoption and fidelity for a larger trial including use of the EHR. BP control in excess of 70% is a remarkable achievement in this setting, exceeding the WHO’s 50% target control by 2040 to avert about 10,000 deaths [14] and the Botswana national goal of 60% BP control by 2025 [13]**.** Recruitment reached 94% of PWH and HTN, adoption (a primary outcomes) of our adapted EHR was low. Therefore, it is likely that uptake of two of our three major strategies (provider training and treatment partner training and support) largely contributed to the success of our study. There was a higher adherence to our main strategies in the smaller clinic compared to the large clinic, likely due to a better provider to participant ratio. We were able to attain greater than 70% BP control, with minimal effect on HIV viral load suppression rates among patients who were in active HIV care at 12 months. We used an implementation research framework (RE-AIM) in this setting to support measurement of effectiveness as well as implementation outcomes.

Our baseline (pre-implementation) HTN control (47.2%) is higher than an earlier meta-analysis of six studies of PWH in sub-Saharan Africa [36], recently published studies in urban Uganda [37], Nigeria [38], and Botswana [12] with control rates of 13.4%, 9.3%, 24.4%, and 41.7%, respectively. In contrast, a South African cross-sectional study of 827 PWH and HTN in Western Cape observed higher BP control (74.1%) [39]. Compared to our study, the South African study [39] had a younger mean age (38.4 years) than ours (54 years), and fewer gaps in the social determinants of health (e.g., education (85.4% vs. 37.6% with ≥ primary education/grade 7), employment (56.1% vs. 35.9%), compared to our study. They [39] also had a lower proportion of PWH with overweight/obesity (43.9% vs 58.3%). Lower levels of education and employment potentially contribute to lack of knowledge about HTN risk factors such as diet, alcohol and physical activity, while high BMI is a well-known risk factor of uncontrolled HTN [14]. This finding underscores the need for BP control strategies that consider the social determinants of health, for instance, by using trained treatment partners to deliver peer level repetitive education to a population with lower levels of education and address other factors such as diet and exercise.

Our strategies and interventions were effective in improving HTN control in two HIV clinics at the end of a 12-month follow-up. Similarly, high levels of HTN control were observed in the Ugandan study, establishing a control of 74.1% at 6 months follow up and intervention [37] which remained relatively stable at 72% at the 21 months assessment [40]. Although the Uganda study [37, 40] and our current study report similar rates of control, the Ugandan study had a greater change in BP control (approximately 65%) 6–21 months later [37, 40], compared to the 24% observed in our study. In addition, our reported improvements in SBP (10.3 mmHg) and DBP (8.5 mmHg) from baseline to 12 months are lower than the improvements in the Ugandan study which reported a SBP and DBP drop of 25.1 mmHg and 12.6 mmHg respectively at 12-months [40]. These large gains in BP drop occurred in the Ugandan setting due to very high baseline uncontrolled BP (as compared to our Botswana study). Notably, their strategies overlapped with ours, and included health education, medication adherence, and lifestyle counseling; routine HTN screening; task shifting of HTN treatment; evidence-based HTN treatment protocol; consistent supply of HTN medicines free to patients; and inclusion of HTN-specific monitoring and evaluation tools. Despite our collective improvements, BP control remains below 80% in both the Botswana and Uganda setting, indicating a need for robust dissemination and implementation science studies to increase BP control rates to match those for HIV. Nevertheless, HIV-HTN integration proved beneficial for HTN control in both settings.

Given that there were high rates of those on BP medications and the correct treatment for that matter, low adherence rates probably contributed to low BP control at baseline. Specific strategies that were associated with the success of our interventions include a high reach and uptake of enrollment into the study (94%), a high completion of provider and treatment partners training per protocol (strategy fidelity) resulting in adoption of medication prescribing, diet, and exercise counseling. This may have improved patient education on HTN and adherence to medications and lifestyle modifications. Additionally, receiving comprehensive and continued CVD care at our study site’s HIV clinic during the follow up period vs the disjointed care at the same clinics prior to our study (baseline) also contributed to our study ‘success.

There was a notable and persistent gap in the adoption by providers of use of all of our EHR adaptations. We hypothesize that the slightly high EHR use at baseline may have been driven by the excitement of the adaptations at the beginning which were introduced during the training, but this momentum may have worn out overtime. As reported in separate metanalyses of EHR use in SSA [41, 42], other possible reasons for this decline and lack of sustained adoption include suboptimal existing infrastructure, slow internet connectivity, frequent power cuts and EHR downtime. Furthermore, the need to simultaneously enter data into both paper records and the EHR systems in our setting and study is similar to other studies, this probably burdened the already short-staffed healthcare workers and perpetuated resistance to the EHR change [41]. There are a limited number of studies in LMICs evaluating the role of EHR in disease monitoring and medication prescription, particularly on integrated care [42]. One of the few publications on HIV/HTN integration using EHR was a Zambian cross-sectional study of a national EHR system with over one million PWH in existence since the early 2000s, which reported that only 13.5% of PWH and hypertension had ≥ 2 recorded blood pressure readings and only 8.9% PWH and hypertension had an anti-hypertensive medication recorded in their EHR [43].

There was also a low adoption of cardiovascular disease risk factor (CVDRF) evaluation similar to with e-prescribing rates, for potentially similar reasons. As described above, more studies are needed to understand why CVD risk stratification for early treatment CVDRFs is not done in this setting. We believe that, in part, more education is required for locally validated CVDRF prediction to be achieved. Considering the REPRIEVE trial results, published after our pilot study had ended, showing that PWH with a low-to-moderate risk of CVD who received a statin had a lower risk of major adverse cardiovascular events [44], the findings support the need for healthcare providers to undertake CVDRF stratification to inform statin prescription given that supplies of statins may not be available to everyone in sub-Saharan Africa (at least for now). Despite this limitation, there was a significant improvement in the e-documentation of cardiovascular risk factor counselling, coupled with an increase in CVDRF knowledge. Our results showed that training healthcare providers, patients and community-based treatment partners increased patients’ knowledge about CVDRF within 12 months.

Despite our study’s success, 27 participants who had discontinued follow up for various reasons were possibly no longer receiving HIV care within the public healthcare system as assessed through the national EHR system. Possible reasons for this attrition include factors unrelated to the study such as loss of life, seeking alternative care through private providers due to personal reasons, or study-related factors. The latter may represent an unintended harm of InterCARE and could be due to factors such as discomfort with study procedures or requirements and stigma. HIV associated stigma may have been exacerbated by receiving HTN care in HIV specific clinics. Future studies should carefully document unintended effects of their implementation strategies for integrated health service delivery. We note that our HIV retention rate of 262 participants (90.3%) at 12-months was still higher than the retention rate in most SSA HIV programs [45] at 75.0% after 12-months follow-up. However, our facility/clinic based retention rate is lower than the prior local study [46], which had a 98% retention rate at the end of the 12 months follow up involving active tracking and tracing including home visits by clinic staff and counselors. InterCARE did not conduct home tracing which may explain why lost-to-follow-up may be higher for this clinic-based study. Among those who stayed in the study until the end of follow-up, HIV viral load suppression rates were similar. This high retention rate and preserved viral suppression rate in our study is consistent with a recent large, pragmatic cluster RCT of > 6000 PWH, INTE-AFRICA [47], which showed that integrated care for people with diabetes, hypertension or both, resulted in a high retention rate without adversely affecting HIV viral suppression.

The limitations of our study are as follows. First, our pilot study's use of convenience sampling and the relatively small sample size potentially limits its generalizability to diverse populations. This may have introduced bias towards improving BP control if only motivated participants were willing to enroll in our study. Second, the absence of a control group makes it difficult to account for unmeasured bias and confounding that could have influenced the BP control and other outcomes. Moreover, since the study only focused on PWH, relevance and implications of the effect of our intervention strategies in the general population is limited. Third, we did not evaluate participants' adherence to blood pressure medications or lifestyle modification in this pilot study, which is crucial for understanding the true impact of our intervention. Fourth, pre-implementation activities may have impacted our initial results, as providers may have started implementing the new strategies immediately after they were trained, prior to participant enrolment. However, given our experience with healthcare providers seeking booster training plus high staff turnover, it is unlikely that training pre-implementation would have significantly impacted our results. Fifth, there was a low adoption of our EHR adaptations, impacting on integrated documentation of medical care. Sixth, we acknowledge that there were no other provider outcomes (besides fidelity) that were directly reported because mechanistically most of the outcomes we reported with the RE-AIM framework were influenced by providers. Moreover, limited resources to conduct the study could not allow us to obtain additional information to report RE-AIM outcomes for each of the three interventions separately. However, we believe that the current RE-AIM outcomes that report on participants’ follow up provide a combination of key outcomes for each of the three intervention strategies. Future studies may consider exploring RE-AIM outcomes for each intervention strategies to facilitate mechanism mapping. Seventh, we also acknowledge the fact that 11.0% of participants discontinued follow up/care, due to lack of interest, transferring care or discontinuation of HIV care, with only 4 of these having documentation of viral load (all of which were undetectable). This could have resulted in retention bias if patients with uncontrolled BP were more likely to be lost to follow up or were more likely to be retained. However, evidence from previous studies has demonstrated that patients lost to follow up are less likely to be adherent to medication [48], making the latter possibility less likely. Lastly, given the nature of our study, we were not directly involved in entering patient data into the EHR system nor we were involved in its validation. We allowed the caring clinician teams to continue doing this routinely, but encouraged EHR use and the adoption of our EHR adaptations for CVD risk stratification. Nevertheless, given the consistency of the baseline HTN control and HIV viral load suppression rates in this study compared to previous publications [12, 15], we believe the data entered was reliable and generalizable. Despite these limitations, our study provides preliminary evidence supporting the effectiveness and safety of our implementation integration strategies for controlling hypertension among PWH.

## Conclusion

The pilot study of the InterCARE strategy in Botswana showed promising results for integrating hypertension care in HIV-care clinics. Of our three major interventions, there was adoption of only two, provider training and use of treatment partners, highlighting the value of hybrid studies measuring effectiveness and implementation outcomes. Our study demonstrated potential for long-term maintenance of the intervention, with sustenance of treatment partners and ongoing support for patients at 12 months. Our findings offer valuable insights for sustainability and scaling up of HTN care integration within HIV programs, not only in Botswana but also in similar settings. Further research is needed to assess the long-term effectiveness and sustainability of this integrated care model and to examine any unintended effects on the HIV program. This supports the need for a large implementation and effectiveness trial.

### Supplementary Information

Supplementary material 1.

## Data Availability

This study is in compliance with the NIH Public Access Policy, which ensures that the public has access to the published results of NIH funded research. All results have been (and will be made) available from final peer-reviewed journal manuscripts (including this one) via the digital archive PubMed Central upon acceptance for publication.

## Abbreviations

BP: Blood Pressure
PWH: People with HIV
HIV: Human Immunodeficiency Virus
HTN: Hypertension
PWH and HTN: People with HIV and hypertension
CVD: Cardiovascular diseases
NCD: Non-communicable disease
ART: Antiretrovirals Therapy
IDCC: Infectious Diseases Care Clinic
BCPP: Botswana HIV Combination Prevention Project
MoH: Ministry of Health
WHO: World Health Organization
OPD: Outpatient department
HDFQ: Heart Disease Fact Questionnaire
REDCap: Research Electronic Data Capture;
HER: Electronic health records
CFIR: Consolidated Framework for Implementation Research
RE-AIM: Reach, Effectiveness, Adoption, Implementation, Maintenance
